# The Relationship Between Food Insecurity and Mental Health Among Syrians and Syrian Refugees Working in Agriculture During COVID-19

**DOI:** 10.3390/ijerph22040549

**Published:** 2025-04-02

**Authors:** Clara Calia, Afnan El-Gayar, Ann-Christin Zuntz, Shaher Abdullateef, Esraa Almashhor, Liz Grant, Lisa Boden

**Affiliations:** 1 School of Health in Social Science, University of Edinburgh, Edinburgh EH8 9AG, UK; 2 The Roslin Institute, Royal (Dick) School of Veterinary Studies, University of Edinburgh, Edinburgh EH25 9RG, UK; aelgaya@ed.ac.uk (A.E.-G.); lisa.boden@ed.ac.uk (L.B.); 3 Social Anthropology, School of Political and Social Science, University of Edinburgh, Edinburgh EH8 9LD, UK; ann-christin.zuntz@ed.ac.uk; 4 Syrian Academic Expertise, Gaziantep 27200, Turkey; shaher.a@sae-afs.org (S.A.); esraa.m@sae-afs.org (E.A.); 5 Global Health Academy, University of Edinburgh, Edinburgh EH8 9AG, UK; liz.grant@ed.ac.uk

**Keywords:** food insecurity, refugees, mental health, syndemic

## Abstract

The COVID-19 pandemic has disproportionately impacted vulnerable populations, such as internally displaced Syrians and Syrian refugees (SSRs) in Middle Eastern host countries, through a syndemic interplay of health, social, and economic challenges. Movement restrictions disrupted their livelihoods resulting in increased food insecurity. A mixed-methods approach was used to address the research question: “What is the relationship between Food Insecurity (FI), Mental Health (MH), and COVID-19 among displaced SSRs working in agriculture”? One hundred SSR participants working in agriculture were recruited from northern Syria and neighbouring countries to participate in a Household Survey. The survey data were analysed using correlation and regression analysis. Additionally, interviews with Household Survey researchers were conducted and thematically analysed. Increasing food insecurity was significantly correlated with worse mental health outcomes among SSR participants (*r_s_* = −0.24, *p* = 0.018). No moderation effects were found with COVID-19 measures or household responses to the pandemic. However, smaller food portions and storing food were positively correlated with poor mental health and food insecurity. The COVID-19 pandemic exacerbated food insecurity and mental health challenges among displaced Syrians and refugees, particularly during Ramadan in 2020, highlighting the compounded effects of overlapping crises and the need for further research into resilience strategies.

## 1. Introduction

The COVID-19 pandemic was predominantly characterised as a public health event and disease mitigation and control measures were adopted accordingly [1,2]. However, the subsequent analysis on pandemic preparedness and response measures recognised the multidimensional, concurrent and overlapping nature of social and economic threats (including war, political instability and food insecurity) and the impact these had on effective pandemic responses in different countries. We now know that the cumulative and amplifying effect of these threats created a syndemic; syndemics examine how social and health conditions develop, how they interact and the underlying factors driving these interactions [3]. The term combines “synergy” and “epidemics”, reflecting the concept that diseases do not occur in isolation. Instead, population health is often shaped by the interplay of various factors—such as climate change or social inequality—that contribute to multiple health issues disproportionately affecting certain groups [4]. COVID-19 was not only a public health emergency of international concern, but also triggered by, and became the trigger for multiple structural inequalities. The interventions to contain the pandemic resulted in multiple unintended consequences including severe food insecurity and poor mental health (Mental Health is defined by the World Health Organization (WHO 2022) as “a state of well-being in which an individual realises his or her own abilities, can cope with the normal stresses in life, can work productively and fruitfully, and is able to make a contribution to his or her community”—available at https://www.who.int/news-room/fact-sheets/detail/mental-health-strengthening-our-response (accessed on 2 August 2024)), particularly in vulnerable groups such as externally and internally displaced populations [5,6,7,8,9].

A key methodological challenge in applying syndemics theory lies in capturing the dynamic and multidimensional interactions between social, environmental, and biological factors over time. For example, mental health and food security share a complex relationship, which has been further intensified by the impacts of COVID-19. Studying the interplay between food insecurity, mental health outcomes, and COVID-19 requires longitudinal data that track these variables simultaneously within affected populations. However, such data are often incomplete, inconsistent, or unavailable in resource-limited settings, making it difficult to demonstrate causality and the complexity of syndemic systems.

The Syrian conflict, which began in 2011, resulted in the internal displacement of more than 7.2 million people [10] and an estimated 6.6 million cross-border displaced Syrians. In absolute numbers, Türkiye hosts the majority of Syrian refugees (3.6 million in 2020) [10], followed by Lebanon and Jordan. In 2020, there were approximately 5.6 million registered Syrian refugees in Iraq, Jordan, Lebanon, and Türkiye [11]. This mass exodus has placed immense economic and social pressure on host countries [12,13]. In Lebanon, many Syrian refugees live in informal tent settlements and struggle to afford basic necessities [14,15]. Jordan hosted over 1.3 million Syrians according to the Jordanian government [16] (or 618,000 according to the United Nations High Commissioner for Refugees (UNHCR)), while Iraq recorded 244,760, a lower number compared to other neighbouring countries [17]. In Türkiye and Jordan, about 80% of Syrians residing in rural and urban areas face significant challenges, including inadequate living conditions, harassment from host communities, and limited access to essential resources such as education and food [12,18].

Syrians living in Syria and other countries in the Middle East had varying experiences during the COVID-19 pandemic due to important differences in economic and political stability within and between countries [19]. Lebanon, for example, implemented early measures such as school closures and social distancing to curb the spread of COVID-19 [19]. Syria’s response was delayed as it was heavily reliant on support from the UNHCR and other non-governmental organizations (NGOs), with limited access to personal protective equipment (PPE) and testing [19].

Many countries, particularly those hosting large numbers of forcibly displaced persons, faced considerable challenges in responding to the pandemic due to limited resources and inadequate infrastructure [20]. Due to the vulnerable status of refugees and issues within Syria in implementing measures to combat the spread of COVID-19, SSRs are vulnerable to worsening conditions and at risk of not being included in global and local responses to COVID-19 [21,22].

### Food (In)security and Mental Health

Food insecurity is defined by the U.S. Department of Agriculture (USDA) as “a household-level economic and social condition of limited or uncertain access to adequate food” [23]. In practical terms, this often means that affected individuals must rely on cheaper, less nutritious food, reduce their overall food intake, and live with the constant uncertainty of whether they will be able to afford food in the future. Prior to COVID-19, food insecurity was already a critical issue affecting the daily lives of Syrians. The ongoing conflict in Syria, subsequent changes in migration patterns, and policies which resulted in the exclusion of refugees from formal labour markets have had significant long-term economic and social effects and have severely destabilised Syrian food security [24,25].

**Figure 1:**
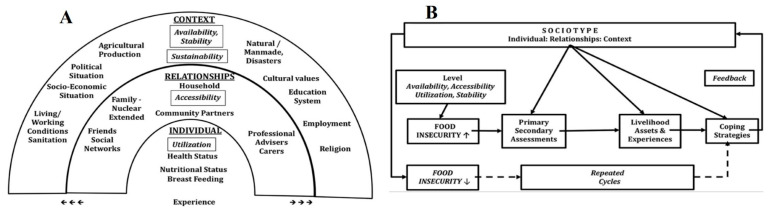
The sociotype ecological framework regarding food insecurity and coping strategies: (**A**) Each domain is indicated with an underlined title. Italics indicate food security dimensions and within each domain there are elements that may relate to coping strategies, preventing FI or stressors that may contribute; (**B**) The involvement of the sociotype in dynamic assessment of FI stressors and coping strategies (From [30]).

The ‘Sociotype Ecological Framework’ explores FI coping strategies by analysing how individuals, their relationships, and their broader context contribute to either sustaining or alleviating FI [26]. Figure 1 illustrates the three interconnected domains: individuals, their relationships, and their wider context, highlighting their interactions and how coping strategies are perceived by those experiencing FI. Peng et al. (2018) [26] proposed a feedback loop within this framework, where events at the contextual level influence the individual, and individual-level actions, in turn, affect the broader context (Figure 1B). The Sociotype Ecological Framework’s feedback loop can be applied to FI issues affecting Syrians and SSRs. The conflict in Syria destabilised the country and its national food security (context), which subsequently impacted individual food security [26]. As individuals and families sought refuge in neighbouring countries, the influx of refugees placed significant strain on the economic and social support systems of host nations, leading to increased FI rates [26]. However, this perspective may oversimplify the situation by overlooking the influence of social and economic policies enacted in response to refugee migration.

The relationship between food insecurity and mental health is well established [27,28] and has been observed in Syrian communities worldwide, including Norway, Turkey, and the United States of America [29,30,31,32]. The protracted nature and outcomes of the ongoing conflict mean that Syrians are predisposed to negative mental health experiences due to the exacerbation of pre-existing mental disorders, emergence of new concerns, and issues related to adaptation to their current circumstances [33]. However, the cross-sectional nature of these observational studies has made it difficult to conclusively determine directionality. Studies suggest that the relationship between mental health and food insecurity is likely bi-directional in any setting, not only in the humanitarian setting [34,35,36,37], but this is difficult to evidence conclusively in cross-sectional studies [37,38,39,40,41].

Due to the nature of the COVID-19 pandemic, there were a number of rapid needs assessments on food insecurity and livelihoods for Syrian refugees, but limited research has been conducted among Syrians to investigate the interplay between food insecurity, mental health, and COVID-19. In this study, we aim to fill this gap by investigating the research question “What is the Relationship Between FI, MH and COVID-19 Among SSRs Working in Agriculture?”. We want to highlight the dynamic nature of these domains within a culturally sensitive setting and gain a deeper insight into its multidimensional effects on vulnerable populations like SSRs.

## 2. Materials and Methods

### 2.1. Study Design

We used a mixed-methods approach utilising data from a Household Survey and interviews with data collectors [5,42,43]. The Household Survey was derived from a multidisciplinary project led by the principal investigator Professor Lisa Boden from the University of Edinburgh: From the FIELD (April–July 2020), an urgent COVID-19 research call project funded by the Scottish Funding Council’s Global Challenges Research Fund. The project—embedded within The One Health FIELD Network (For more information: https://onehealthfieldnetwork.com/ (accessed on 4 August 2024))—was designed to capture the experience of displaced Syrian agricultural workers during the COVID-19 pandemic during the summer of 2020. For this project, a bottom–up approach was employed, emphasising collaboration and co-creation with Syrian academics affiliated with the Council for At-Risk Academics (CARA) (CARA is a UK-based civil society organisation that supports displaced academics, including through a (now defunct) Syria Programme for Syrian academics, mostly located in Türkiye (https://www.cara.ngo/what-we-do/caras-syria-programme (accessed on 4 August 2024)), and Lebanese research partners.

This approach aimed to ensure that the research framework and outcomes were directly informed by the lived experiences, cultural knowledge, and expertise of those most affected. By prioritising input from these stakeholders, the project sought to foster culturally relevant methodologies, amplify local perspectives, and promote equitable partnerships, ultimately ensuring that the findings and interventions were grounded in the realities of the communities they aimed to serve. The topics of the survey included socio-demographics; sanitation and cleanliness; mental health; physical health; food; livestock; work; individual and community coping strategies; and gender. Interviews were conducted with researchers who had collected the Household Survey data, to provide additional detail, context, and prospective concerning aspects of FI and MH during COVID-19.

### 2.2. Participant Recruitment

#### 2.2.1. Household Survey

Inclusion criteria for the survey required participants to have worked or work in agriculture. Recruitment was primarily conducted by CARA Syria academics. These researchers were mostly Syrian (as well as one Jordanian and two Lebanese), and except for one Syrian, were based in the study countries, so directly affected by the pandemic.

We were able to recruit participants from isolated rural areas and communities that lack connections to aid organisations as researchers utilised professional contacts and their personal kinship networks together with the application of snowballing techniques. Participants were identified in Iraq, Jordan, Lebanon, Syria, and Türkiye, and 20 people were selected to take part in the study, from each country. All participant data were anonymised and stored digitally in password-protected files. Participants were paid the equivalent of GBP 10 for participation either through phone credit or in cash. Cash payments were limited due to the pandemic. Prior to participating in the survey, all participants received a written information sheet and consent form in Arabic via WhatsApp. To ensure clarity, interviewers also sent a voice message explaining the study’s terms in simple language. Additionally, at the start of each interview, an oral consent protocol was followed to confirm participants’ understanding of the research purpose. When needed, interviewers provided contact details for local NGOs that could offer support. Due to the then ongoing movement restrictions and some participants’ limited literacy, we conducted interviews via WhatsApp. WhatsApp, an end-to-end encrypted messaging platform, was chosen for its widespread use among SSRs for maintaining familial and social connections, as well as for its data security features. With participants’ consent, phone conversations were recorded to ensure the accurate capture of data during interviews.

#### 2.2.2. Researcher Interviews

Researchers who collected data for the Household Survey were approached and invited for a semi-structured online interview. Because most of the researchers were also Syrian refugees and had experience with SSR communities outside the Household Survey, they provided valuable insights that enhanced the depth of survey analysis. Their shared ethnic and cultural familiarity allowed them to interpret meaning beyond the literal responses, capturing elements such as pauses, emphasis on specific words, and tonal variations during discussions. These non-verbal cues often convey implicit meanings, emotions, or cultural contexts that might otherwise be overlooked in a purely textual analysis. Additionally, researchers with an insider perspective can identify idiomatic phrases, and contextual references that contribute to a more comprehensive understanding of the data. This deeper level of analysis is particularly important in qualitative research, where meaning is often shaped by social and cultural contexts. By incorporating these interpretive skills, they were able to contribute valuable cultural context to the discussion and ensured a more accurate and culturally sensitive representation of participants’ perspectives rarely accessible to researchers based in the West.

### 2.3. Materials

#### 2.3.1. Household Survey

The questionnaire was developed as a pilot study. It included validated tools to measure food insecurity (Coping Strategies Index—CSI) and mental health (Warwick–Edinburgh Mental Wellbeing Scale—Seven-Item Version, SWEMWBS) [44,45,46,47]. The SWEMWBS was selected for its validity among Arabic-speaking populations. The local collaborators determined that its non-intrusive and positively framed wording made it well suited to the sensitive humanitarian context [46]. The scale addresses emotional and functional dimensions, tracking mood fluctuations and the impacts of daily stressors effectively.

The CSI was slightly adapted to fit the survey’s context, adhering to guidelines from the 2008 Field Manual produced by the Cooperative for Assistance and Relief Everywhere and the World Food Programme. Additionally, two COVID-19-specific measures (C19_a_ and C19_p_) assessed participants’ awareness and preparedness. C19_a_ evaluated the availability and type of COVID-19 information SSRs received, as well as its sources, while C19_p_ assessed participants’ preparedness, including access to face masks and hand sanitiser.

The survey consisted of 96 questions, with COVID-19-related data captured in questions 46–48 (C19_a_) and 55–56 (C19_p_). Data analysis was conducted using SPSS 27 (2020) to ensure rigorous examination of responses.

#### 2.3.2. Researcher Interviews

Five researchers, who collected data for the Household Survey, were interviewed in English, via Zoom 5.0.1 (2020)/WhatsApp 2.20.108 (2020), and their interviews were recorded with their consent. NVivo12 [48] software was used to analyse the interviews. Coding and thematic analysis were conducted in Microsoft Word, with Miro (a mind mapping tool) being used to develop final visualisations of themes.

### 2.4. Data Analysis

#### 2.4.1. Household Survey

The raw dataset was cleaned, and responses were removed that were either not demographic in nature (Q2–17) or concerning elements of the primary research question, i.e., MH (Q35–41), FI (Q57–68) or COVID-19 (Q46–48, Q55–56, Q85 and Q86). Questions regarding the location were simplified to the participants’ current country. The monthly rent was converted into USD to simplify comparison between countries using historical exchange rates. Missing scores for CSI (FI) and SWEMWBS (MH) were replaced with mean averages for each question. Conversion for SWEMWBS scores was applied following guidelines and CSI scores were weighted according to its manual [49]. Correlation and regression analyses were conducted to establish and determine the relationship between FI, MH, C19_a_ and C19_p_. Household/societal responses to COVID-19 were used in Spearman’s correlation to identify relationships with FI, MH, C19_a_ and C19_p_. Significant correlations with both FI and MH were further investigated using moderated hierarchical regression.

#### 2.4.2. Researcher Interviews

Thematic analysis was conducted on interview transcripts per Braun and Clarke (2006). Thematic analysis was chosen in favour of interpretive phenomenological analysis as it allowed for patterning across all participants and flexibility in the coding and analysis process [49,50]. Thematic analysis also suited the interview focus of recounting the experiences of survey participants second-hand [51]. Familiarity with researcher interviews revealed that the impact of other economic and/or political factors on SSRs should be considered in the analysis. The thematic analysis was therefore conducted in a theoretical/deductive manner in order to investigate the second-hand experiences of field work researchers and to describe the experience of research participants regarding mental well-being; food insecurity; and the impact of COVID-19 and other economic/political issues on SSRs. The text was described in initial codes, and these were later refined to have consistency between the interviews. From these codes, patterns were identified and were assigned to themes. Themes were refined and checked against initial coding before being defined and named.

## 3. Results

### 3.1. Quantitative Analysis (Household Survey)

One-hundred survey participants (75 males, 25 females) from Iraq, Jordan, Lebanon, Syria and Türkiye gathered data for approximately 568 people. Only MH scores were normally distributed, all other measures did not satisfy parametric assumptions (including normality). Therefore, all correlations were conducted using Spearman’s rank correlation coefficient.

#### 3.1.1. Correlation Analysis

The average score for CSI measuring FI was 18.85 (*SD* = 4.06), and the average score for MH using the SWEMWBS was 25.58 (*SD* = 4.25), indicating moderate well-being among participants. A statistically significant correlation was found between FI and MH (*r_s_* = −0.24, *p* = 0.018), illustrating that higher FI was associated with worse MH.

Spearman’s rank correlations were used to assess whether C19_a_ and C19_p_ moderated the relationship between FI and MH. A positive correlation was found between worse C19_p_ and FI (*r_s_* = 0.29, *p* = 0.004). Two significant correlations were found at the household level: not storing food (*r_s_* = 0.21, *p* = 0.038) and having smaller meal portions (*r_s_* = −0.35, *p* < 0.001) were associated with worse FI. No significant correlations were observed between FI and societal COVID-19 responses (Appendix A, Table A2 and Table A3). At the household level, storing food was correlated with a better MH (*r_s_* = −0.21, *p* = 0.039), while having smaller portions was correlated with worse MH (*r_s_* = 0.364, *p* < 0.001). No significant correlations were found between MH and the societal COVID-19 responses (Appendix A, Table A2 and Table A3). Storing food and having smaller portion sizes were also found to be consistently significant predictors of MH and FI in the models.

#### 3.1.2. Regression Analyses

Linear regression analysis revealed a significant model and confirmed that FI was a significant predictor of MH (*F*(1,98) = 0.83, *p* = 0.030), explaining 4.7% of the variance in MH scores (*b* = 0.23, *t*(−2.199), *p* = 0.030) (Appendix A, Table A1). However, the hierarchical regression analysis showed that neither C19_a_ nor C19_p_ significantly moderated the relationship between FI and MH (Table 1).

Upon conducting a moderated hierarchical regression, no significant moderation was found, despite having smaller portion sizes being a consistently significant predictor in all models (Table 2). Only Step 1 indicated a significant model (*F*(3,96) = 4.83, *p* = 0.004).

### 3.2. Qualitative Analysis (Researcher Interviews)

**Figure 2:**
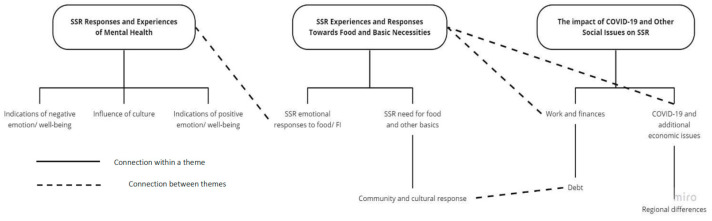
Mind map displaying the three themes (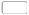), their emerging sub-themes (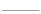) and how they interact (- - - - -). The figure depicts how sub-themes relate to larger themes and the potential interaction between themes as well. Note how the theme “SSR Responses and Experiences of Mental Health” is connected to the theme “SSR Experiences and Responses Towards Food and Basic Necessities” through sub-theme “SSR responses to Food/FI”.

Deductive thematic analysis was conducted on the transcripts and additional emails from interviews with researchers who collected data from the Household Survey. Three main themes emerged (Figure 2):

#### 3.2.1. SSR Responses and Experiences of Mental Health

**Figure A1:**
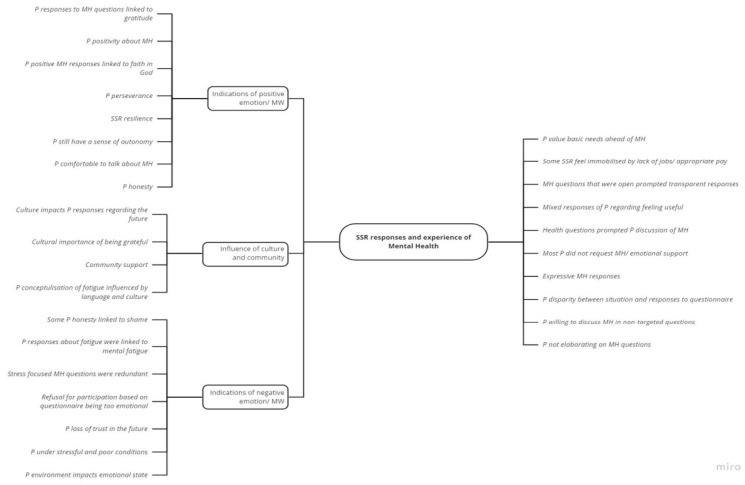
Mind map of the theme ‘SSR Responses and Experience of Mental Health’.

Sub-themes collate the positive and negative aspects of MH indicated by researchers in these interviews and how culture may have an influence and included (Appendix A, Figure A1):

- Indications of positive emotion/well-being: Many SSRs expressed MH in positive terms, often linked to religious beliefs and a sense of resilience. However, there was a noted loss of trust in the future.
- Indications of negative emotion/well-being: Despite the general positive outlook, there were concerns about the future, especially regarding children’s well-being, reflecting the broader societal situations impacting the MH of SSRs. Similar sentiments were reported by long-term displaced Syrian refugees in Lebanon, who experienced anxiety, depression, hopelessness, sadness, and diminished self-worth.
- Influence of culture and community: Cultural and religious beliefs shaped how SSR participants expressed their mental health. A notable influence was the role of religious faith, which fostered a generally positive outlook despite challenges. Many participants emphasised gratitude towards God as a coping mechanism when faced with adversity, exemplified by expressions such as “thank God for what we have” (Interview 1). This perspective reflects the protective role of faith in providing comfort, hope, and meaning during times of hardship.

#### 3.2.2. SSR Experiences and Responses Towards Food and Basic Necessities

**Figure A2:**
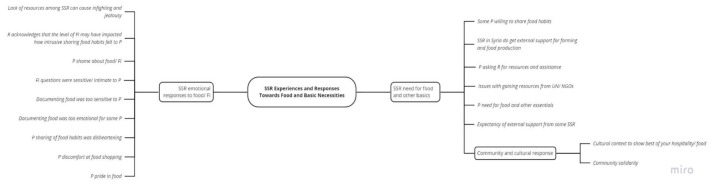
Mind map of the theme ‘SSR Experiences and Responses Towards Food and Basic Necessities’.

This theme incorporates the experience participants had with necessities, in particular food. Codes also were clustered under three sub-themes (Appendix A, Figure A2)

- Emotional responses to FI/food: SSRs often expressed emotions an emotion-based approach when discussing food habits such as shame or distress. Nevertheless, others expressed pride in the nature of their cooking.
- SSR feed for food and other basics: This sub-theme highlighted the lack of availability of resources and SSR reliance on external bodies for support.
- Community and cultural responses: A sense of solidarity and camaraderie within the community. SSRs relied on community-based food distribution practices, and extended networks to mitigate food insecurity (such as relying on neighbours).

#### 3.2.3. The Impact of COVID-19 and Other Social Issues on SSRs

**Figure A3:**
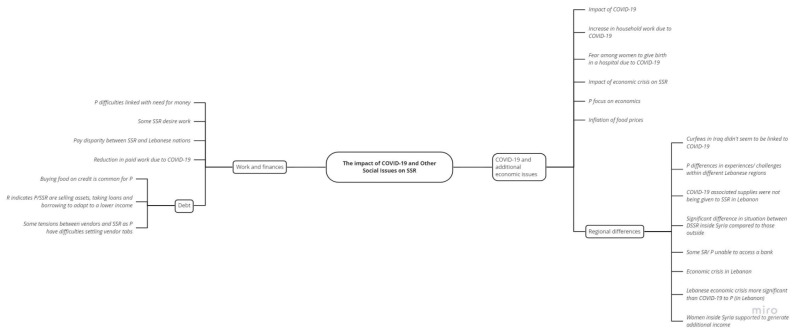
Mind map of the Theme ‘The Impact of COVID-19 and Other Social Issues on SSR’. Note. Shown are the codes that contribute to each sub-theme and how these relate to the overall theme. Codes are shown in italics.

This theme captured the impact that COVID-19 and current economic/financial issues had on participants (Appendix A, Figure A3).

- COVID-19 and additional economic issues: The impact of the pandemic was often overshadowed by pre-existing economic challenges, such as inflation. The pandemic merely exacerbated these difficulties.
- Work and financial changes: The impact of COVID-19 and the existing economic challenges that COVID-19 exacerbated were keenly observed through changes in work available to SSRs. Many Syrian refugees lost their jobs due to disruptions in agricultural supply chains and pandemic-related restrictions, exacerbating already precarious working conditions shaped by limited labour rights and legal ambiguity. While work was disrupted for many, some displaced people continued working as the beginning of the pandemic coincided with the start of the agricultural season in the Middle East.
- Debt: SSR communities were able to exhibit coping mechanisms, including resource-sharing, informal debt networks or getting a loan from an NGO to counteract the impact of the current economic issue. However, many respondents reported having nothing left to sell and no one to borrow from, indicating that their communities’ ability to cope with shocks had already been eroded before the pandemic.
- Regional differences: Some interviews referred to the current economic crisis in Lebanon as playing a significant role in impacting the lives of SSRs.

## 4. Discussion

The COVID-19 pandemic impacted everyone, but affected people disproportionately. Populations like SSRs who had already been made vulnerable because of conflict, climate change or financial inequities were made even more vulnerable [3]. We aimed at providing a glimpse into the early impacts of the pandemic on SSRs beyond disease at an intersection of FI and MH, emphasising the syndemic effects that exacerbate the vulnerabilities of these populations [8]. Through applying a mixed-methodological approach, this research provides insights into how FI and MH are interrelated and how SSRs’ resilience and coping mechanisms can inform potential long-term trends, future interventions and support strategies.

Our analysis revealed that the mental health and attitudes of SSRs were generally positive. However, the interplay between FI and MH among SSRs cannot be fully understood without considering the broader cultural and contextual factors that shape these experiences. In our study, cultural norms, religious beliefs and structural constraints significantly influenced how participants perceived, experienced and responded to FI and MH challenges. The positive state of MH was largely attributed to religious influences. This aligns with existing research suggesting that religious faith can provide a psychological buffer against distress, fostering hope and meaning during crises [52,53,54]. These practices have been associated with promoting positive well-being and served as a coping mechanism which warrants consideration in future research and interventions [55,56,57,58]. However, while religious gratitude plays a significant role in fostering mental well-being, its impact appears less robust compared to dispositional gratitude [56]. Furthermore, reliance on religious gratitude may also discourage help-seeking behaviours and limit engagement with formal mental health services, particularly when distress is normalised as part of religious endurance [55].

While adults reported good personal mental health, they highlighted deteriorating MH in their children, such as experiencing nightmares. The disparity within the answers raises important questions about the cultural and social factors influencing self-disclosure. Adults may have found it less stigmatising or easier to discuss their children’s MH struggles than their own, potentially reflecting cultural norms around self-presentation and emotional vulnerability. MH challenges also surfaced indirectly in the health section of the survey. These issues were often indirectly revealed through questions about physical health, current circumstances, or the environment. This aligns with studies indicating that in collectivist societies, psychological distress is more likely to be discussed in relational or physical terms rather than as an individual pathology [33].

Conducting our research during Ramadan was a particularly influential cultural factor and allowed us to capture the intersection of food insecurity, mental health, and religious observance within a pandemic setting. Religious fasting during Ramadan directly affected food consumption and preparation practices among participants, adding complexity to their experiences of food insecurity and resilience strategies. Our findings suggest, that while religious coping mechanisms were present, they were also challenged by social distancing measures. Traditional Ramadan gatherings, which are central to fostering a sense of community and solidarity, were largely disrupted by COVID-19 social distancing measures. This limitation not only altered dietary practices but also impacted participants’ sense of belonging and social support, highlighting the intersection of religious observance, community dynamics, and the broader effects of the pandemic on psychosocial well-being.

The Household Survey revealed significant levels of FI among participants. SSR coping mechanisms are consistent with the sociotype ecological framework, which emphasises leveraging relationships and contextual resources to manage FI [26]. It is crucial, however, to avoid romanticising “community-based resilience pathways” when addressing FI. While these informal support systems were essential, they were also fragile. Borrowing from family and neighbours had diminished due to prolonged economic hardship. This reality highlights a critical gap: although formal interventions often fail to complement or build upon collective practices [18], the erosion of these networks means that many individuals could not rely on collective coping mechanisms. When external interventions overlook the autonomy and resilience patterns of SSRs, they risk disempowering communities rather than supporting them [59,60,61].

While the data initially suggested the potential for a bi-directional relationship between FI and MH—where FI could both result from and contribute to poor MH—triangulating these findings with other data points challenges this interpretation. An important contextual attribute lies in the lack of rights for Syrians as refugees and workers across host countries, exposing them to exploitative labour conditions while largely excluding them from healthcare access. Many SSR participants were subject to policies that excluded them from formal labour markets, restricting economic stability and reinforcing food insecurity. For example, in Lebanon, employment restrictions forced many Syrians into informal, unstable agricultural work, often without labour protections or social security [43]. These structural exclusions resulted in chronic economic stressors that compounded mental health challenges. Without a rights-based approach to addressing Syrian displacement such as formalising labour contractors’ roles and fostering local employment forums, celebrating community resilience risks masking the systemic failures of host countries and the international community [62,63]. However, this study could not determine whether these responses were a result of poor mental health or contributed to it, suggesting a potential bi-directional relationship between FI and MH in the context of COVID-19 [34,35,37]. Such findings would be best supported by a follow-up study considering the longitudinal effects of COVID-19 on FI and MH.

Our results indicate that COVID-19 information was not translated into action to prevent the spread of disease, potentially because of a general lack of resources among SSRs [64,65,66,67]. This lack of resources, coupled with high FI, highlights the challenges SSRs faced in accessing appropriate PPE and maintaining their livelihoods during lockdowns [68]. The results of this study align with broader research indicating that syndemic effects exacerbate the vulnerabilities of SSRs [3,69]. This position, however, does not consider that a relationship between FI and COVID-19 preparedness (C19_p_) may be driven by a fear of developing COVID-19 rather than a lack of resources alone [70]. As global food prices soared due to disruptions in food supply chains and economic recession [71,72,73,74], SSRs experienced worsening financial circumstances and increased FI [73,74].

Additionally, interviews indicated a lack of resources from governments and NGOs being given to SSRs and stress may have been exacerbated by limitations on the movement of aid and inequality in social support given to citizens when compared with SR [19,75,76,77,78]. Access to formal mental health services was highly limited. In many host countries, mental health support for refugees was either unavailable or provided only to those within formal refugee camps, leaving those in urban and informal settlements underserved [79]. The lack of culturally and linguistically appropriate mental health services further exacerbated barriers to care.

In contrast, some countries like Jordan provided MH support to SR within camps during the pandemic [79], demonstrating the positive outcomes of the interventions. However, access to such support remains complex, with social protection often reserved for those who have previously contributed to the host country’s social system prior to the pandemic aligning with pre-pandemic trends regarding refugees which had been prevalent in lower-/middle-income countries (LMICs) [80]. Our participants were residing outside of formal refugee camps, predominantly in informal agricultural settlements or urban areas. As a result, they would not have been eligible for this support, which highlights an important gap in MH support.

This study highlights the urgent need for understanding the cultural and contextual dimensions of FI and MH within vulnerable populations. Future research should explore the long-term effects of cultural coping strategies, particularly how religious gratitude and collective resilience interact with psychological distress over time. Additionally, policies aimed at addressing refugee well-being should incorporate both economic inclusion measures and community-based mental health support, ensuring that interventions align with the lived realities of displaced populations. Drawing on the insights provided by SSR participants and local researchers, we propose the following policy and practice recommendations:

Strengthen community-centred food security interventions: Although SSR communities have developed collective coping strategies, these networks are under strain. Humanitarian actors and policymakers should co-design food security interventions that align with these existing social practices rather than impose top–down models that may undermine them. For example, adapting multi-purpose cash assistance and e-voucher schemes to support collective purchasing and food-sharing arrangements would increase both food access and community cohesion.

Recognise and integrate cultural and religious coping strategies in mental health support: Participants’ reliance on religious faith and expressions of gratitude as protective mechanisms indicates the importance of culturally sensitive mental health interventions. Mental health programs should engage faith leaders and community advocates, incorporate religious frameworks where appropriate, and offer psychosocial support that respects and amplifies these cultural values.

Formalise refugee labour rights and protect agricultural workers: Given that food insecurity among SSRs is closely linked to precarious, informal agricultural labour, the legal recognition and protection of Syrian refugees as workers is essential. This includes the right to work formally, protection from exploitation, and access to fair wages and social security benefits. Formalising the status of displaced agricultural workers would not only improve household food security but also contribute to the economic stability of host countries’ agricultural sectors.

Bridge the gap between policy and lived experience through participatory approaches: Our study demonstrates that local researchers and SSR community members possess critical insights into effective coping mechanisms and barriers to food security and mental health. Future interventions and policies should adopt participatory approaches that involve SSRs in decision-making processes—from needs assessments to intervention design and evaluation. This will ensure that aid is locally relevant, culturally appropriate, and sustainable.

Address structural causes of food insecurity and mental health challenges: Finally, addressing food insecurity and poor mental health requires confronting the structural drivers of vulnerability, including economic exclusion, lack of legal status, and inadequate access to healthcare and social services. Long-term solutions must involve regional collaboration to ensure equitable rights and protections for displaced populations, moving beyond emergency responses to sustainable inclusion policies.

By centring SSR voices and experiences, these recommendations aim to move beyond descriptive analysis to inform actionable strategies that can mitigate the compounded effects of food insecurity and mental health challenges in displaced populations.

## 5. Conclusions

COVID-19 has significantly impacted the FI and MH of SSRs, primarily through social factors such as economic disruption and limitations on aid and governmental support. While COVID-19 did not moderate the relationship between FI and MH in this study, further research is needed to explore the role of emotional responses to food insecurity and their influence on MH outcomes. Additionally, future research should consider the protective role of religious gratitude in maintaining relatively higher MH scores among SSRs.

Applied research is crucial to developing FI interventions that align with collective coping strategies, while also promoting self-esteem and resilience within this population. The factors affecting SSRs are multifaceted and cannot be comprehensively addressed in a single study. Therefore, continued research is essential, and a mixed-methods approach is recommended to capture the nuanced experiences and data.

Moreover, it is important that future research be conducted in a bottom–up, collaborative manner, involving Syrian academics to ensure that SSR input is integrated at all levels of the research process—not just as participants. This approach will enhance the relevance and applicability of interventions and findings in addressing the complex challenges faced by SSRs.

### Limitations

While this study provides important insights into the relationship between FI, MH, and COVID-19 among SSRs working in agriculture, we fully acknowledge several key limitations. 

First, the limited generalisability of our findings must be underscored. Our sample, recruited through convenience and snowball methods, focused on an extremely marginalised subgroup of SSRs working in agriculture, many of whom were living in informal settlements or rural areas with limited access to formal aid. As such, while our data provide valuable insights into this often-overlooked population, they cannot be generalised to all Syrian refugees in the Middle East or beyond. Nonetheless, the shared experiences of structural vulnerabilities, economic exclusion, and disrupted livelihoods identified in this group may reflect broader patterns affecting displaced populations living in precarious conditions, and therefore, our findings can inform tailored interventions for similarly situated communities.

Second, the cross-sectional nature of our study inherently limits causal interpretations. Although our findings highlight significant associations between FI and MH, and point to COVID-19 as a critical amplifying factor, the temporal direction of these relationships remains unclear. It is plausible that worsening FI contributes to poorer MH, but it is also possible that pre-existing MH difficulties exacerbate the experience of FI. Moreover, the overlap of COVID-19 with long-standing socio-economic challenges complicates efforts to disentangle these effects. We therefore emphasise the need for future longitudinal research that could illuminate the evolving nature of these relationships over time and assess the long-term impacts of the pandemic on FI and MH.

Finally, while COVID-19 did not emerge as a statistically significant moderator of the FI–MH relationship in our quantitative analysis, our qualitative findings highlight the pandemic’s important role as an exacerbating factor. COVID-19 lockdowns, movement restrictions, and supply chain disruptions compounded existing vulnerabilities, limiting access to food, income, and social support. Importantly, participants and researchers emphasised that COVID-19 was not experienced in isolation but rather as one layer of crisis within an ongoing syndemic context of displacement, poverty, and conflict. Therefore, while the direct moderating role of COVID-19 may not have been detected statistically, its structural and indirect effects on both FI and MH are substantial and merit greater recognition.

We also acknowledge that data were collected during the summer of 2020, an early phase of the COVID-19 pandemic. Since then, societal, economic, and health contexts may have evolved, potentially influencing the applicability of conclusions drawn. However, our findings highlight that food insecurity and mental health challenges among displaced Syrians were not solely a consequence of the pandemic but were deeply rooted in pre-existing structural vulnerabilities, such as economic exclusion, limited labour rights and inadequate social protections. While a study conducted at a later stage of the pandemic might capture shifts in specific pandemic-related stressors—such as changes in employment patterns, aid availability, or vaccine access—it is likely that the core issues of food insecurity and mental health distress remain persistent due to broader economic and political factors. Moreover, our thematic analysis suggests that, even in the early pandemic phase, many Syrian refugees viewed COVID-19 as one crisis among many, rather than a singular defining event. Future research employing a longitudinal approach would be valuable in assessing how the interplay between food insecurity, mental health, and economic instability has evolved post-pandemic. Such studies could examine whether pandemic recovery efforts, shifts in humanitarian aid, or inflationary pressures have altered coping strategies and well-being outcomes among displaced populations.

## Figures and Tables

**Table 1:** Moderated Hierarchical Regression results for Food Insecurity, COVID-19 Awareness and Preparedness on Mental Health.

Variable	B	95% CI for B		SE B	β	R^2^	∆R^2^
		LL	UL				
Step 1						0.09	0.092 *
Constant	29.25 ***	24.38	34.12	2.45			
C19_p_	−1.09	−2.35	0.17	0.64	−0.18		
C19_a_	0.18	−0.12	0.47	0.15	0.12		
FI	−0.19	−0.40	0.03	0.11	−0.18		
Step 2						0.09	0.001
Constant	29.39 ***	24.39	34.39	2.52			
C19_p_	−1.12	−2.41	0.17	0.65	−0.18		
C19_a_	0.17	−0.13	0.47	0.15	0.11		
FI	−0.19	−0.41	0.03	0.11	−0.18		
FI × C19_a_	0.11	−0.71	0.93	0.42	0.03		
Step 3						0.09	0.000
Constant	29.32 ***	24.14	34.49	2.61			
C19_p_	−1.10	−2.44	0.23	0.67	−0.18		
C19_a_	0.17	−0.14	0.48	0.16	0.11		
FI	−0.19	−0.41	0.03	0.11	−0.18		
FI × C19_a_	0.13	−0.74	0.99	0.44	0.03		
FI × C19_p_	−0.05	−0.91	0.81	0.43	−0.01		
Step 4						0.09	0.000
Constant	29.29 ***	24.07	34.51	2.63			
C19_p_	−1.09	−2.46	0.29	0.69	−0.18		
C19_a_	0.18	−0.14	0.50	0.16	0.12		
FI	−0.19	−0.41	0.03	0.11	−0.18		
FI × C19_a_	0.10	−0.89	1.08	0.49	0.02		
FI × C19_p_	−0.07	−1.02	0.88	0.48	−0.02		
C19_a_ × C19_p_	0.06	−1.01	1.13	0.54	0.01		
Step 5						0.09	0.001
Constant	29.73 ***	23.74	35.72	3.02			
C19_p_	−1.16	−2.64	0.31	0.74	−0.19		
C19_a_	0.15	−0.21	0.51	0.18	0.10		
FI	−0.19	−0.42	0.032	0.11	−0.19		
FI × C19_a_	0.09	−0.90	1.01	0.50	0.02		
FI × C19_p_	−0.10	−1.08	0.88	0.49	−0.03		
C19_a_ × C19_p_	−0.03	−1.25	1.19	0.62	−0.01		
FI × C19_a_ × C19_p_	0.14	−0.77	1.05	0.46	0.05		

CI = confidence interval; LL = lower limit; UL; upper limit; FI = food insecurity; C19_a_ = COVID-19 awareness; C19_p_ = COVID-19 preparedness. * *p* < 0.05. *** *p* < 0.001.

**Table 2:** Moderated Hierarchical Multiple Regression Results for Food Insecurity, COVID-19 Household Responses on Mental Health.

Variable	B	95% CI for B		SE B	β	R^2^	∆R^2^
		LL	UL				
Step 1						0.13	0.131 **
Constant	24.80 ***	19.08	30.51	2.88			
FI	−0.10	−0.31	0.12	0.11	−0.09		
SP	2.33 **	0.58	4.08	0.88	0.27		
SF	−1.14	−2.78	0.50	0.83	−0.13		
Step 2						0.14	0.009
Constant	25.44 ***	19.58	31.29	2.95			
FI	−0.12	−0.34	0.10	0.11	−0.11		
SP	2.12 *	0.32	3.91	0.90	0.25		
SF	−1.21	−2.86	0.44	0.83	−0.14		
FI × SP	−0.47	−1.40	0.46	0.47	−0.10		
Step 3						0.14	0.001
Constant	25.43 ***	19.55	31.31	2.96			
FI	−0.12	−0.34	0.10	0.11	−0.12		
SP	2.13 *	0.32	3.93	0.91	0.25		
SF	−1.22	−2.88	0.44	0.84	−0.14		
FI × SP	−0.49	−1.43	0.45	0.47	−0.10		
FI × SF	0.13	−0.71	0.97	0.42	0.3		
Step 4						0.14	0.000
Constant	25.45 ***	19.52	31.39	3.00			
FI	−0.12	−0.34	0.10	0.11	−0.12		
SP	2.12 *	0.30	3.94	0.917	0.25		
SF	−1.22	−2.88	0.45	0.84	−0.14		
FI × SP	−0.48	−1.45	0.48	0.49	−0.10		
FI × SF	0.11	−0.80	1.03	0.46	0.03		
SP × SF	−0.04	−0.95	8.71	0.46	−0.01		
Step 5						0.14	0.001
Constant	25.53 ***	19.54	31.51	3.01			
FI	−0.13	−0.36	0.10	0.12	−0.12		
SP	2.06 *	0.19	3.94	0.94	0.24		
SF	−1.11	−2.94	0.72	0.92	−0.13		
FI × SP	−0.50	−1.48	0.47	0.49	−0.10		
FI × SF	0.14	−0.80	1.07	0.47	0.03		
SP × SF	−0.01	−0.94	0.92	0.47	−0.00		
FI × SP × SF	0.14	−0.82	1.11	4.9	0.03		

CI = confidence interval; LL = lower limit; UL; upper limit; FI = food insecurity; SP = smaller portions; SF = storage of food. * *p* < 0.05. ** *p* < 0.01. *** *p* < 0.001.

## Data Availability

The dataset(s) supporting the conclusions of this article are available on a platform supported by the University of Edinburgh; DataSync.

## Abbreviations

The following abbreviations are used in this manuscript:

CARA	Council for At-Risk Academics
CSI	Coping Strategy Index
FI	Food Insecurity
LMICs	Lower-/Middle-Income Countries
MH	Mental Health
MPCA	Multi-Purpose Cash Assistance
NGO	Non-Governmental Organisation
PPE	Personal Protective Equipment
SRs	Syrian Refugees
SSRs	Syrians and Syrian Refugees
SWEMWBS	Warwick–Edinburgh Mental Wellbeing Scale
UNHCR	United Nations High Commissioner for Refugees
USDA	The United States Department of Agriculture
WHO	World Health Organization

## Appendix A

**Table A1:** Spearman’s Rank Coefficient Correlation Matrix.

Variable	*n*	*M*	*SD*	1	2	3	4
6. MH	100	25.58	4.25	-			
7. C19_p_	100	2.44	0.69	−0.16	-		
8. C19_a_	100	8.76	2.81	0.09	−0.09	-	
9. FI	100	18.85	4.06	−0.24 *	0.29 **	0.15	-

Note: MH = Mental Health; C19_p_ = COVID-19 preparedness; C19_a_ = COVID-19 awareness; FI = Food Insecurity * *p* < 0.05. ** *p* < 0.01.

**Table A2:** Spearman’s rank correlation coefficient for Household Responses to COVID-19 against Mental Health, COVID-19 Awareness/Preparedness and Food Insecurity.

Variables	*n*	*M*	*SD*	1	2	3	4	5	6	7	8	9
1. MH	100	24.58	4.25	-								
2. C19_p_	100	2.44	0.69	−0.158	-							
3. C19_a_	100	8.76	2.81	0.086	−0.092	-						
4. FI	100	18.85	4.06	−0.237 *	0.287 **	0.146	-					
5. No change	100	1.96	0.20	0.004	−0.116	0.031	0.086	-				
6. Storage of food	100	1.45	0.50	−0.206 *	0.198 *	0.028	0.208 *	−0.226 *	-			
7. Having smallerportions	100	1.40	0.49	0.364 **	−0.191	0.096	−0.352 **	−0.250 *	−0.123	-		
8. Cleaning more than normal	100	1.18	0.39	−0.077	0.213 *	0.124	0.099	−0.436 **	0.361 **	0.043	-	
9. No visiting with family/ friends	100	1.03	0.17	−0.079	0.175	−0.035	−0.042	−0.862 **	0.194	0.215 *	0.375 **	-

Note: MH = Mental Health; C19_p_ = COVID-19 preparedness; C19_a_ = COVID-19 awareness; FI = Food Insecurity; * *p* < 0.05. ** *p* < 0.01.

**Table A3:** Spearman’s rank correlation coefficient for Societal Responses to COVID-19 against Mental Health, COVID-19 Preparedness/ Awareness and Food Insecurity.

Variables	*n*	*M*	*SD*	1	2	3	4	5	6	7	8	9	10
1. MH	100	24.58	4.25	-									
2. C19_p_	100	2.44	0.69	−0.158	-								
3. C19_a_	100	8.76	2.81	0.086	−0.092	-							
4. FI	100	18.85	4.06	−0.237 *	0.287 **	0.146	-						
5. No change	100	1.97	0.17	−0.088	0.015	0.046	0.152	-					
6. Information distribution	100	1.57	0.50	−0.074	0.077	−0.258 **	0.027	0.084	-				
7. Organisation of food items	100	1.54	0.50	−0.086	0.150	0.012	0.017	−0.045	0.049	-			
8. Staying home	100	1.11	0.31	0.084	−0.047	0.143	−0.094	−0.313 **	−0.017	0.068	-		
9. No social gatherings	100	1.08	0.27	0.066	−0.070	0.024	−0.070	−0.164	0.033	0.198 *	0.485 **	-	
10. Mosque announcements	100	1.70	0.46	0.055	0.005	−0.326 **	−0.126	0.013	0.181	−0.079	0.091	0.032	-

Note: MH = Mental Health; C19_p_ = COVID-19 preparedness; C19_a_ = COVID-19 awareness; FI = Food Insecurity; * *p* < 0.05. ** *p* < 0.01.
